# Sensitivity of caloric test and video head impulse as screening test for chronic vestibular complaints

**DOI:** 10.6061/clinics/2017(08)03

**Published:** 2017-08

**Authors:** Raquel Mezzalira, Roseli Saraiva Moreira Bittar, Marcia Maria do Carmo Bilécki-Stipsky, Cibele Brugnera, Signe Schuster Grasel

**Affiliations:** I Departamento de Otorrinolaringologia, Faculdade de Medicina FMUSP, Universidade de Sao Paulo, Sao Paulo, SP, BR; II Clínica de Otorrinolaringologia do Instituto Penido Burnier, Campinas, SP, BR

**Keywords:** Caloric Test, Video Head Impulse Test, Vestibular Ocular Reflex, Vertigo

## Abstract

**OBJECTIVE:**

This study compared the results of the caloric test with those of the video head impulse test obtained during the same session and evaluated whether the former can be used to screen for non-acute vestibular dysfunction.

**METHODS:**

A total of 157 participants complaining of dizziness with vestibular characteristics of varying durations and clinical courses completed the caloric test and video head impulse test.

**RESULTS:**

Significantly more caloric test results than video head impulse test results were abnormal.

**CONCLUSIONS:**

The results of the caloric test and video head impulse test are distinct but complement each other. Within our sample, the caloric test was more sensitive for vestibular dysfunction. Therefore, the video head impulse test is not a suitable screening tool of the vestibular system in patients with chronic complaints.

## INTRODUCTION

The caloric test (CT) is the most accepted method of evaluating peripheral vestibular function. However, its limitation is well known: it only stimulates the lateral semicircular canal at low frequencies 1, whereas everyday head movements occur at higher frequencies and along all three planes 2.

The video head impulse test (vHIT) is the computerized version of the head impulse test (HIT) described by Halmagyi and Curthoys in 1988 3. The HIT uses small-amplitude and high-acceleration movements around the vertical axis while the patient fixates on a stable target.

Gain is the magnitude of the eye movement that results from the head impulse. When the vestibular ocular reflex (VOR) is normal, the patient is able to keep the eyes fixed on the target, and the eye movement shows the same angular velocity in the same plane and opposite direction as the head movement. In this case, the gain is 1. When the compensating ocular movement is insufficient, a central command will launch a corrective saccade to bring the eye back to the target. When the corrective saccade occurs after the head movement has stopped, it is called an *overt* saccade. An overt saccade indicates a VOR alteration due to an abnormality of the stimulated canal. During vestibular compensation over time, this saccade will be substituted by a saccade that occurs during the head movement called a *covert* saccade. Covert saccades are likely a part of the central compensation that anticipates the final eye position according to the expected head movement. It has been proposed that cervical proprioceptors deliver this information or that the function of the peripheral organ is suppressed during the head impulse 4,5,6. The HIT is most important for cases of acute dizziness and when abnormal, is highly suggestive of a peripheral vestibular lesion. Along with spontaneous nystagmus and skew eye deviation, the HIT has higher sensitivity than magnetic resonance imaging for detecting stroke within the first 48 hours 7. The vHIT is more sensitive to saccades, particularly covert saccades 8, than the HIT, and the VOR measurement and registration are more reliable and can be used during patient follow up. Currently, this test is considered as a fundamental tool for the physical examination of patients with acute dizziness.

Because the vHIT is important for the evaluation of dizziness, the question arises whether vHIT can substitute the bithermal CT for the diagnosis of vestibular disorders. Several studies have compared vHIT and CT results among patients with vestibular complaints. According to the literature, the vHIT cannot serve as a substitute for the CT as a screening tool for patients with vestibular dysfunction because these tests measure different aspects of the VOR 2,9,10.

One explanation for the discrepant results between these tests is the anatomic and physiologic background of the VOR. The receptor of the angular VOR is the crista ampullaris, which contains type I and type II cells. This structure receives regular and irregular neural afferent discharges. Type I cells are in the middle of the crista and decode high frequency and fast acceleration head movements. These cells are connected to irregular afferent fibers. Type II cells are found in the periphery of the crista and decode low frequency and low acceleration head movements. They are connected to regular afferent fibers.

Thus, regular vestibular afferent fibers show a relative higher gain at low frequencies, whereas irregular fibers demonstrate higher gain at high frequencies 11. Both vHIT and the CT are used to evaluate unilateral VOR but at different frequencies: the vHIT evaluates frequencies above 5 Hz using rapid and short head impulses, whereas caloric irrigation activates lower frequency bands (0.003 Hz) 12.

Nevertheless, the vHIT and CT differ not only in frequencies but also in the way the stimulation occurs. During the vHIT, a rapid head impulse generates a physiologic endolymphatic flow. In contrast, the caloric stimulus induces an endolymphatic flow via a temperature gradient. The CT stimulates the inner ear independent of gravity 13.

The aims of the current study are as follows:

To compare the results of the CT and vHIT obtained during the same session and

To evaluate the viability of the vHIT as a screening tool for non-acute vestibular dysfunction.

## MATERIALS AND METHODS

This multicenter cross-sectional cohort study was conducted in accordance with the standards of the ethical committee (CAPPesq) of the Hospital das Clinicas, University of Sao Paulo Medical School (approved on March 2nd, 2016, number 1433854) and complies with the Helsinki Declaration of 1975.

### Study population

This casuistic study included 157 participants (88 women and 69 men) attending the Neurotology Outpatient Clinic of Hospital das Clinicas, University of Sao Paulo Medical School and the Otolaryngologic Clinic Penido Burnier, Campinas, SP. The mean age of the participants was 49±16.7 years. The common clinical complaints included vestibular dizziness of a variable duration and clinical course. All of the patients received standard clinical testing including medical history, otolaryngologic and cranial nerve examinations, static and dynamic balance tests (Romberg and Fukuda), coordination tests (diadochokinesis with alternating pronation, forearm supination and index finger–nose tests) and complete videonystagmography. After patient selection, the CT and vHIT were applied to the sample.

### Protocol

We analyzed and compared the CT and vHIT results of patients with dizziness complaints.

Caloric stimulations were performed with water at 30°C and 44°C or with air at 24°C and 50°C. Responses to caloric stimuli are similar using air at these temperatures 14. Both ears were stimulated separately with a 5-minute interval between tests. We used an Interacoustics VN415 (Denmark) and ICS Chartr 200 Otometrics (Denmark) for these tests. The Jongkees formula was applied to calculate the unilateral weakness (UW) and gain asymmetry (GA) based on the angular velocity values of the slow phase obtained via the warm and cold stimulations of the right and left ears.


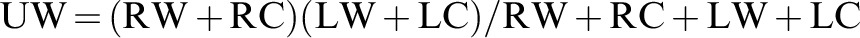



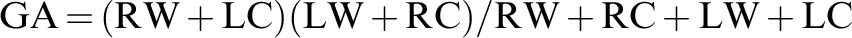


### Jongkees Formula: RW=right warm, RC=right cold, LW=left warm, LC=left cold

UW and GA values above 20% were considered abnormal. The angular velocity values of the slow component were also analyzed. We considered values under 5 degrees/second to represent hypofunction and values above 50 degrees/second to represent hyperfunction 3,14,15. A morphologic or qualitative analysis of the CT recording was performed.

**Figure 1:**
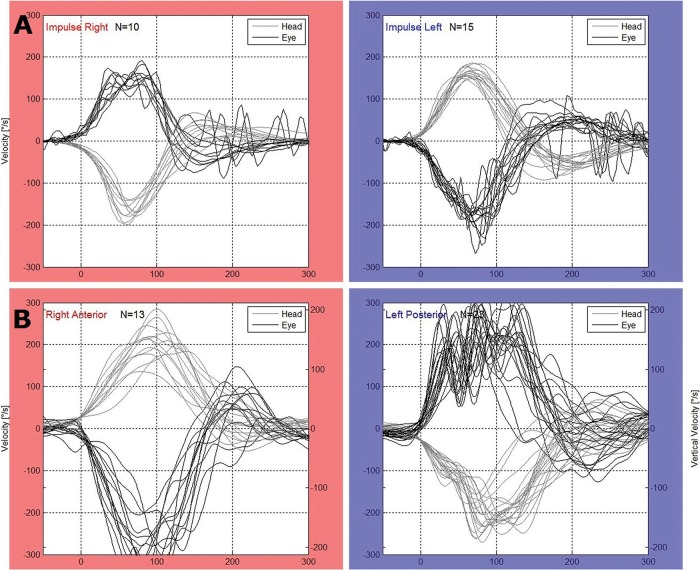
vHIT chart. A: normal test, B: velocity decay, low gain and corrective saccades on the left side (see Cam vHIT Interacoustics).

For the vHIT, we used the Eye See Cam vHIT Interacoustics and ICS Impulse Otometrics. The horizontal VOR was evaluated. Therefore, unpredictable manual head impulses were applied at approximately 20 degrees with a mean velocity of 150 degrees/second and a mean acceleration of 1,000 to 2,500 degrees/second^2^ in the horizontal plane with the patient fixing his or her eyes on a target placed 1 m in front of them. At least 20 adequate impulses were applied to the right and left sides for each test. The test was considered as abnormal when the VOR gain was lower than 0.8 2,8,16. The presence of overt or covert saccades was also considered (Figure 1).

### Statistical analysis

The participants’ mean (standard deviation) age was calculated. Fisher’s exact test was used to analyze the clinical data. The results are shown in 2×2 contingency tables that applied a two-tailed test. Significance was set at α=0.05.

## RESULTS

Our sample was composed of 157 participants (88 women and 69 men) aged 49±16.7 years.

A total of 113 abnormal CT and 41 abnormal vHIT tests were found among the sample (Table 1). The absolute value of the angular velocity of the slow phase (AVSP), the UW and GA of the CT, and the gain of the vHIT was considered abnormal. The CT identified significantly more abnormal results than the vHIT (Fisher’s exact test, *p*=0.008). When we compared the results between the tests, the proportion was 3:1. Therefore, we had 3 abnormal CT results for every abnormal vHIT result.

The vHIT revealed abnormal UW values in 19 participants and normal values in 53 normal participants (*p*=1). A more detailed analysis of the UW values showed that in the UW range between 20 and 40%, eight patients exhibited an abnormal and 33 a normal vHIT test. Regarding UW values >40%, we found that 11 patients showed abnormal results, and 20 had normal vHIT results (*p*=0.18). No significant difference was found in either case. No significant difference in vHIT gain was observed among normal and abnormal CT results.

Comparing the angular velocity values of the slow phase below 5 degrees (hypofunction) with the vHIT gain, we found 24 patients with hypofunction and 16 abnormal vHIT results. Of the 133 patients with normal function, 25 exhibited an abnormal vHIT test. The CT was more sensitive than the vHIT (*p*=0.0001). These data are shown in Table 2.

The comparison between the angular velocity value of the slow phase below 5 degrees (hypofunction) and the corrective (overt and covert) saccades was statistically significant (*p*=0.0001). Conversely, when the angular velocity values of the slow phase exceeded 50 degrees, they were not related with saccades (*p*=0.5; Tables 3 and 4).

The qualitative analysis of the CT (post caloric rhythm, morphologic nystagmus alterations and fixation index absence) revealed no significant correlation with the vHIT gain (*p*=0.30) or vHIT saccades (*p*=0.57).The vHIT did not show abnormalities when the CT demonstrated qualitative alterations. These data are shown in Tables 5 and 6.

## DISCUSSION

Undoubtedly, the vHIT plays an important role during the bedside physical examination of patients with acute dizziness. It is a relevant diagnostic tool for acute vertigo and is currently considered essential in emergency room units in which dizzy patients are treated. Alterations in VOR gain are key to differentiating between stroke and peripheral lesions. The current study focused on elements that can be evaluated in vestibular diseases.

The CT is the most commonly used vestibular function test in clinical practice, and our major objective was to compare these VOR gain results with those measured using the vHIT in patients with vestibular disorders. We questioned whether the vHIT could be used instead of the CT as a screening tool for vestibular dysfunction. If the results of these tests are correlated, then the vHIT can be used as a screening tool for patients with balance disorders to identify those who require a CT. The ability of the vHIT to document semicircular canal function over a brief time period would be a clear advantage over the CT.

Although other studies have described a higher prevalence of vHIT gain alterations during the first five symptomatic days 8, we did not consider symptom duration in this study, nor did we describe the vestibular diagnosis of our patients because we believed that our sample was not large enough.

Overall, we found 113 abnormal CT results and 41 abnormal vHIT results, indicating 3 abnormal CT results for every abnormal vHIT result. Thus, the probability of diagnosing a vestibular dysfunction via CT was 72%, whereas that via vHIT was 26%. In our sample, the CT was ultimately more sensitive for diagnosing vestibular diseases.

When we analyzed the relationship between CT abnormalities and vHIT gain, we observed that abnormal post caloric responses were significantly more frequent than vHIT gain alterations (<0.0085). Only 5 participants with normal CT results presented with an abnormal vHIT result, whereas 77 showed abnormal CT results but normal vHIT results. These data are consistent with the findings of Bell et al. 2 who compared both tests and did not find any correlation between them.

Some authors have reported a linear correlation between CT asymmetry and vHIT gain. To observe an abnormal vHIT result, the asymmetry should be near 40% 8,10. When we consider the UW values between 20 and 40% and those >40% compared to the vHIT gain, we did still not obtain a correlation between the measures (*p*=0.1779). Therefore, our results suggest that lateral VOR gain does not predict the degree of lateral semicircular canal paresis. Likewise, we did not observe any correlation between UW, regardless of its value, and vHIT gain alteration.

When we analyzed the relationship between CT hypofunction and abnormal vHIT gain, we found a significant correlation (*p*<0.0001) between low vHIT gain and lack of function of the lateral semicircular canal during the CT 2.

A significant relationship was also found between overt and covert saccades and AVSP values below 5 degrees. In our sample, the likelihood that the vHIT would diagnose vestibular hypofunction was 66%.

However, when we analyzed AVSP values greater than 50% as well as overt and covert saccades, no significant relationship was found. We suggest that the inappropriate standardization of the upper reference limit of the vHIT gain explains this finding 2.

In addition to the quantitative analysis, we performed a qualitative analysis of the CT. The most frequent morphologic abnormality was dysrhythmia, showing major variations of amplitude and frequency without AVSP variation. Because the cerebellum controls the nystagmus amplitude 17, these alterations suggest that this structure is involved. The vHIT findings did not indicate the presence of rhythm abnormalities (*p*=0.1458). This result was not surprising because vHIT gain is related to semicircular canal function, whereas post-caloric rhythm variations reveal the involvement of the central nervous system. We did not find references in the literature regarding this approach; however, these findings suggest that the vHIT is unable to diagnose the morphologic abnormalities observed with the CT.

The differences between the vHIT and the CT are based on the alterations documented by the VOR. Two pathways are involved in VOR activation: fast-and-direct (type I neuron) and slow-and-indirect (type II neuron) pathways. The latter is responsible for storage velocity. Storage velocity describes the characteristic of the vestibular system that maintains the response from the peripheral organ even after hair cell stimulation has ceased. This ability results from the joint activation of the primary and secondary neurons that show different characteristics of activation and depolarization. The direct pathway consists of three neurons and transmits the signal from the semicircular canals directly to the ocular effector muscles without modulation.

The indirect pathway also receives information from the semicircular canals but exhibits an elevated time constant for charge and discharge. In this way, the neurons of the indirect pathway “store” the energy acquired by the peripheral organ stimulation and continue to discharge even after the stimulation has ceased 17. The caloric stimulus is a perfect example of storage velocity. It produces a nystagmus response that begins approximately 20 seconds after the beginning of the stimulation, peaks at approximately 40 seconds and decreases until it disappears at approximately 3 minutes (type II neurons). However, the vHIT response depends on the direct pathway of three type I neurons 18. These tests demonstrate different responses to crista ampullaris stimulation.

As in cochlear disease, the involvement of the semicircular canals can lead to specific frequency dysfunctions: whereas the vHIT represents stimuli at approximately 5 Hz, the CT stimulus is approximately 0.003 Hz. Because these tests evaluate different segments of the semicircular canals, both are necessary for a comprehensive vestibular evaluation 2,8,9,10,11,16,18,19.

The vHIT and CT results are diverse and complement each other. They can be considered tests that describe the tonotopy of the crista ampullaris depending on the stimulation frequency. In our sample, the CT was more sensitive to vestibular dysfunction. Therefore, we do not believe that vHIT is an adequate screening tool of the vestibular system for patients with chronic complaints.

## AUTHOR CONTRIBUTIONS

Mezzalira R and Bittar RS were responsible for the literature review, patient selection, interpretation of the tests and preparation of the manuscript. Bilécki-Stipsky MM was responsible for the literature review and interpretation of the tests. Brugnera C was responsible for the tests executions. Grasel SS was responsible for the statistical analysis and text structuring.

## Figures and Tables

**Table 1:** CT and vHIT test results.

	Normal CT results	Abnormal CT results	
**Normal vHIT**	39	77	116
**Abnormal vHIT**	5	36	41
	44	113	

**Table 2:** CT hypofunction compared with abnormal gain in the vHIT.

	Presence ofcaloric hypofunction	Absence ofcaloric hypofunction	
**Abnormal vHIT**	16	25	41
**Normal vHIT**	8	108	116
	24	133	

**Table 3:** Hypofunction (AVSP<5 degrees) and the presence of overt and covert saccades.

	Overt	Covert	
**AVSP> 5**	10	34	44
**AVSP< 5**	34	20	54
	44	54	

**Table 4:** Hyperfunction (AVSP>50 degrees) and the presence of overt and covert saccades.

	Overt	Covert	
**AVSP>50**	0	2	2
**AVSP<50**	44	52	96
	44	54	

**Table 5:** Comparison between the qualitative alterations of the CT and vHIT gain.

	Normal qualitative analysis	Abnormal qualitative analysis	
**Normal vHIT**	83	33	116
**Abnormal vHIT**	33	8	41
	116	41	

**Table 6:** Comparison between the qualitative alterations of the CT and vHIT saccades.

	Normal qualitative analysis	Abnormal qualitative analysis	
**Absent saccades**	69	27	96
**Present saccades**	47	14	61
	116	41	
